# Supplementary material for: Proteome profile of patients with excellent and poor speech intelligibility after cochlear implantation: Can perilymph proteins predict performance?
Source: PLoS One. 2022 Mar 3;17(3):e0263765. doi: 10.1371/journal.pone.0263765 (PMC8893673; doi:10.1371/journal.pone.0263765)
Supplement: Attachment — Submitted filename: Responds to View Letter_PLOSONE_25-10-21.doc [file pone.0263765.s001.doc]

### Hannover Medical School

Department of Otolaryngology and

Head and Neck Surgery

Carl-Neuberg-Str. 1

D-30625 Hannover

Germany

HNO-Klinik der Medizinischen Hochschule Hannover

**Professor and Chairman:**

Thomas Lenarz, M.D., Ph.D

Fax: +49-511- 532-5558

lenarz.thomas@mh-hannover.de

www.mhh-hno.de

**Normal Consultations:**

Tel.: +49 (0)511-532-3032

Fax: +49 (0)511- 532-8001

Mo-Fr by appointment

**Consultations for Private Patients**

**Prof. Thomas Lenarz, M.D. Ph.D**.:

Tel.: +49 (0)511-532-6562

(Mrs. Schmidt)

Wednesdays by appointment

**Consultant Office Hours:**

Tel.: -49 (0)511-532-6562

(Mrs. Schmidt)

Mon., Tue., Thu. and. Fri.

by appointment

**Subspecialty Office Hours:**

Consultations for Skull Base and Orbital Surgery

(Rolf Salcher, M.D.):

Tel.: +49(0)511-532-3032

Mondays

**Tumor Consultations**

**(Martin Durisin, M.D., Ph.D.):**

Tel.: +49 (0)511-532-3032

Tuesdays

**Consultations for Nasal and Sinus Surgery**

Sleep Medicine, Allergology and Rhinoplastic Surgery

**(Constantin M. Weber, M.D.):**

Tel.: +49 (0)511-532-3032

Thursdays

**Consultations for Disorders of the Middle Ear and Trachea**

**(Magnus Teschner, M.D., Ph.D., MBA):**

Tel.: +49 (0)511-532-3032

Fridays

Hanover Ear Institute

Hörzentrum Hannover:

Karl-Wiechert-Allee 3

D-30625 Hannover

Germany

Tel.: +49(0)511-532-6603

Fax: +49 (0)511-532-6833

dhz.info@mh-hannover.de

www.hoerzentrum-hannover.de

**Prof. Dr. med. Th. Lenarz:**

**Prof. Dr. med. Th. Lenarz:**

OE 6500, Carl-Neuberg-Str. 1, D-30625 Hannover

Ihr Zeichen	Mein Zeichen	Tel.: (+49) 511-532-	Hannover, d. 25.10.21

Fax: (+49) 511-532-5558

**Subject:** PLOS ONE: Edits requested on your submission PONE-D-21-08371R1

Dear Edrian Nim Tolentino and Plos One Team,

I write you regarding your mail of October 25^th^ 2021 “Subject: PLOS ONE: Edits requested on your submission PONE-D-21-08371R1 - [EMID:d5fd962aa91ce111]“ because your issues have already been answered and included in the “Review manuscript” and the “Responds to Reviewers” uploaded on October 15^th^ 2021:

1. In your Methods section, please provide additional information about the participant recruitment method and the demographic details of your participants. Please ensure you have provided sufficient details to replicate the analyses such as: a) a description of any inclusion/exclusion criteria that were applied to participant recruitment, b) a description of how participants were recruited, and c) descriptions of where participants were recruited and where the research took place.

**We responded on October 15^th^ 2021**

**in “Response to Reviewers”:** 3. In your Methods section, please provide additional information about the participant recruitment method and the demographic details of your participants. Please ensure you have provided sufficient details to replicate the analyses such as: a) a description of any inclusion/exclusion criteria that were applied to participant recruitment, b) a description of how participants were recruited, and c) descriptions of where participants were recruited and where the research took place.

***Response:*** *The demografic data of the study participants are depicted in detail in Table 1 – 3. The Tables were now integrated in the running text as required in the style requirements of the journal.*

*Additionally we included following information in the methods section:*

*“The inclusion criteria for perilymph sampling is the planned procedure of a CI surgery. Every patient undergoing CI surgery has the ability to participate in the study/perilymph sampling and to sign the consent form for the study in parallel to the consent form for the CI surgery. The only exclusion criteria is the existence of any infectious disease of the patient or the impossibility of perilymph sampling due to the individual surgical procedure decided by the surgeon. The proteome analysis takes place in the Hanover Medical School.”*

**in “Revision Manuscript”:** 3. Table 1 (line 101-103), Table 2 (line 201-202) and Table 3 (line 203-204) (demographic data) and in “Perylymph Sampling”-section line 112-116

2. Please ensure your Methods and reagents are described in sufficient detail for another researcher to reproduce the experiments described. Specifically, please ensure you have provided a brief description of perlymph sample preparation, or an appropriate reference for standard methods.

**We responded on October 15^th^ 2021**

**in “Response to Reviewers”:** 4. Please ensure your Methods and reagents are described in sufficient detail for another researcher to reproduce the experiments described. Specifically, please ensure you have provided a brief description of perlymph sample preparation, or an appropriate reference for standard methods.

***Response:*** *In the methods section we refer to our previous publication (Proteome Analysis of Human Perilymph using an Intraoperative Sampling Method. Journal of Proteome Research. 2017 Mar 10;acs.jproteome.6b00986; Schmitt et al.). In this publication a detailed description of the sampling method, perilymph sample preparation and analysis is included.*

**in “Revision Manuscript”:** in “Material and Methods” section (“Perilymph sampling” and “Proteomic analysis”) line 106, 107 and 121 and in the “References” section line 483.

In case we addressed in the document “Response to Reviewers” (October 15^th^ 2021) each comment in detail also the Journal requirements including the issues mentioned above, we are hoping the revised version is already suitable for publication in PLOS ONE.

Please let us know in detail if there is still any question.

Yours Sincerely,

Athanasia Warnecke
